# Exploring a Complementary Stress Management and Wellbeing Intervention Model for Teachers: Participant Experience

**DOI:** 10.3390/ijerph18179009

**Published:** 2021-08-26

**Authors:** Stevie-Jae Hepburn, Annemaree Carroll, Louise McCuaig

**Affiliations:** 1 School of Education, The University of Queensland, Brisbane, QLD 4067, Australia; a.carroll@uq.edu.au; 2 Faculty of Humanities and Social Sciences, The University of Queensland, Brisbane, QLD 4067, Australia; l.mccuaig@uq.edu.au

**Keywords:** wellbeing, mindfulness, yoga, stress management, health, teachers, mixed-methods, early career teachers, meditation

## Abstract

Background: work-related stress can have alarming physiological and psychological health impacts, which may permeate into personal and professional contexts. Teachers need to be supported to develop the skills and strategies to effectively identify how stress manifests and how to use simple, practical techniques to manage and reduce the impact of stress. Complementary interventions (CIs) for educators may provide stress management and assist with supporting wellbeing at the individual level. Methods: the convergent mixed-methods study included participant reflections, self-report measures for perceived stress, mindful attention awareness, and subjective wellbeing and biological measures (salivary cortisol levels). Data analysis: inductive thematic analysis and mixed-methods case study design. Results: the participants shared that they experienced benefits in personal and professional contexts, behavioural changes, increased awareness of the impact of stress, and a decrease in the stress response. The participant reflections provided contextual information surrounding the self-report and biological measures. The inferences generated were reflected in both datasets. The findings supported the proposed model linking the mechanisms present in the techniques from the system of yoga and the dimensions of wellbeing. Conclusions: the findings suggest that a CI for educators may provide strategies for supporting wellbeing and assisting with stress management.

## 1. Introduction

Teaching is one of the most rewarding professions, and yet almost a third of teachers in the *Teaching and Learning International Survey* from the Organization for Economic Cooperation and Development [1] question their career choice. According to the Australian Institute for Teaching and School Leadership [2], in 2019 “44% of early career teachers indicated they were not likely to leave classroom teaching in the foreseeable future” [2] (p. 106), 26% were unsure, and 20% indicated they would leave within the first five years of teaching. Based on the increasing evidence that teacher wellbeing has an impact on student wellbeing and achievement [3,4,5], it is not surprising that health promotion for teachers is a growing area. In general, the workplace is becoming a setting for the promotion of preventative health activities and physical and psychological wellbeing [6]. Healthy, creative, motivated teachers inspire, nurture, and support their students to achieve and develop a love of learning, which is why teacher wellbeing is far too important to be overlooked.

Research over the past 40 years has consistently highlighted the importance of programs in the pre-service and early career period that prioritise wellbeing and stress management strategies (e.g., [7,8,9,10,11,12]). The *Initial Teacher Education Data Report 2019* [2] outlined that, in Australia, the most common focus for induction programs reported by early-career teachers (ECTs) was “orientation” (reported by 97% of ECTs that completed an induction program), and 39% reported the induction program had no focus on “teacher wellbeing.” Regarding whether the induction experience supported personal wellbeing, 45% of the ECTs disagreed; however, 68% of ECTs felt they were part of the profession due to the induction process. The present study did not aim to promote a single solution for the multifaceted issue of teacher stress. The aim was to highlight the importance of teacher-level (secondary) interventions [13] and present the argument that the approach must be holistic [7] and focus on promoting integrated (holistic) wellbeing. A primary (organisation)-level intervention [14] was not feasible however the present study allowed for an exploration of the implementation of a complementary intervention (CI) for educators.

### 1.1. Background

For decades, researchers have attempted to identify sources of stress for teachers and investigate the responses to stress for different groups based on demographic and biographical characteristics (e.g., age, gender, teaching experience). Researchers have focused on teachers’ perceptions of stress, job satisfaction, burnout, and coping strategies [15]. The research indicates that teaching is consistently viewed as physically, emotionally, and cognitively challenging across various cultural contexts, even in countries where the profession is well-respected (e.g., Finland) [16]. Compared to other professions, teachers experience high levels of psychological distress, psychological burnout, and fatigue [15,17,18]. In the United Kingdom, teaching was ranked as one of the six most stressful jobs, physically and psychologically, in a comparison of 26 occupations [19]. However, caution is advised when drawing conclusions that teaching is one of the most stressful professions from studies (e.g., [19]) that lack details surrounding the participant sample and the statistical significance [20]. Additionally, inconsistencies can result from differing methodologies and theoretical frameworks underpinning research surrounding teacher stress and wellbeing [15].

Even though there are some inconsistencies in the literature surrounding teacher stress and wellbeing, factors (e.g., organisational predictors such as school climate and mentoring) consistently influence job satisfaction and work-related stress [15]. In addition to perceived stress, sickness absence, increased attrition rates, and early retirement are growing in prevalence in the teaching profession [21]. A recent survey of Australian teachers (*n =* 886) revealed that 50% of the respondents had considered leaving the profession due to stress and dissatisfaction in the previous month, and 77% (*n* = 681) considered their job to be moderately, very, or extremely stressful. ECTs reported significantly higher stress levels and elevated student-related burnout than mid- or late-career teachers, whereas work-related burnout was elevated in both early- and mid-career teachers in comparison to those in their late-career [20].

Early research indicates that those in human services (interpersonal professions) can tend to be emotionally invested in their roles [22] and those working in helping professions can be prone to stress due to idealistic goals [23]. Teachers are potentially more vulnerable to stress and burnout due to the day-to-day care required for their students [15]. It is important to note that the circumstances associated with teaching (classroom-based and school environment) are not the sole basis for teachers’ work-related and perceived stress. An assortment of factors influencing wellbeing and distress (perceived stress) are mediated by the interplay between teachers’ values, skills, coping strategies, and dispositions [12]. However, research indicates that work-related stress and mental disorders connected to work can create serious problems for the professionals and their families, the students in their care, the school community, and the broader economy [24]. An important consideration is that individuals’ wellbeing can influence how they interact and engage with their peers and colleagues [25], which is a critical aspect of the early career period (e.g., professional relationships, mentoring). Establishing relationships is an essential factor in professional identity development [26].

Research indicates that a teacher’s perception of the school climate plays a prominent role in influencing perceived stress [27]. It is important to note that the experiences of school life are influenced and shaped by internal and external factors at both the individual and collective (community) levels. Interpersonal relationships such as, for example, the role of parents and family, influence school climate and culture. As outlined by Cohen and colleagues [28], “one of the fundamentally important dimensions of school climate is relational and involves how ‘connected’ people feel to one another in school”. Positive social relationships are fundamental to psychological wellbeing [29], and individuals may be influenced by their exhausted and stressed colleagues and may be susceptible to emotional contagion [15].

### 1.2. The Early Career Period

It is recommended by the OECD [1] (p. 8) that “the higher risk of attrition of younger and novice teachers deserves attention, given the teacher shortages that a number of countries are facing”. There is often a mismatch between the expectations of ECTs (intrinsic, idealistic motivation) and the reality of the classroom [25,30]. The high attrition rates of ECTs have been linked to dissatisfaction with working conditions (e.g., [1,31]). Not all ECTs are destined to suffer work stress due to individual characteristics, previous experience, and motivation [32]; however, the early career period (one to five years of experience) is viewed as an especially vulnerable time [33].

Work stress is described as a physical and emotional response resulting from the mismatch between job requirements and the abilities and resources available for the individual. It is suggested that there is an impact on the individual emotionally when they appraise themselves as ill-equipped to deal with work stressors [34]. Stressors are determined by the individual’s appraisal of the situation and their perception of their ability to cope. It is recommended that ECTs are provided with the opportunity to reflect, discuss, and connect with experienced colleagues and peers [35]. This process supports the development of coping mechanisms and professional identity formation [9], reduces feelings of isolation [36,37], and creates the environment for peer mentoring, social development, and professional conversations [35,38,39]. ECTs often lack the skills and strategies to establish an emotional balance and manage feelings of helplessness, frustration, worry, and anger [8,40]. Providing the opportunity to connect with others gives ECTs a chance to cope with the emotional labour of teaching [41].

### 1.3. Teacher Wellbeing Research

Wellbeing needs to be studied of its own accord, not simply as the absence of ill-being, and approaches that aim to improve psychological wellbeing must be differentiated; that is, treating disorder must be distinguished from preventing disorder, thereby increasing flourishing [42] furthermore, Huppert [42] outlined that increasing flourishing in a population may serve as a preventative measure and a proactive approach. Previous interventions for educators have focused primarily on mindfulness-based approaches developed for clinical applications and the treatment of stress-related conditions. For a summary of the existing research of mindfulness-based interventions (MBIs) for educators, see [43,44]. Yoga, however, is more often associated with fitness and health promotion [45] and to date, limited studies examine the use of yoga-based techniques for stress management or wellbeing interventions for educators.

There is a broad range of methodologies included in research surrounding teacher stress and wellbeing. Multi-measure studies combine quantitative, cross-sectional survey-type approaches with behavioural and physiological indices of stress, whereas qualitative studies can be influenced by misinformation, retrospective interpretation, and bias [15]. It is argued (e.g., [15]) that to progress the field of research in teacher stress and wellbeing, innovative methodologies are needed. Research concerning the perspectives of individuals entering the profession (e.g., pre-service and ECTs) is lacking as well as longitudinal studies examining sources of teacher stress [37]. Exploration of how stressors relate to the characteristics of the individual teachers (e.g., personality factors) and longitudinal study design was beyond the scope of the present study; however, the study design aimed to make a valuable contribution to the research surrounding ECTs’ stress management and teacher wellbeing more broadly.

The present study aimed to address the following research questions:

1. What were the reported experiences of the participants in the Complementary Intervention (benefits, disadvantages, program content, and application)?
2. How does the data generated through the participant reflections about their experience help explain the quantitative results?

### 1.4. Study Design

**Figure 1:**
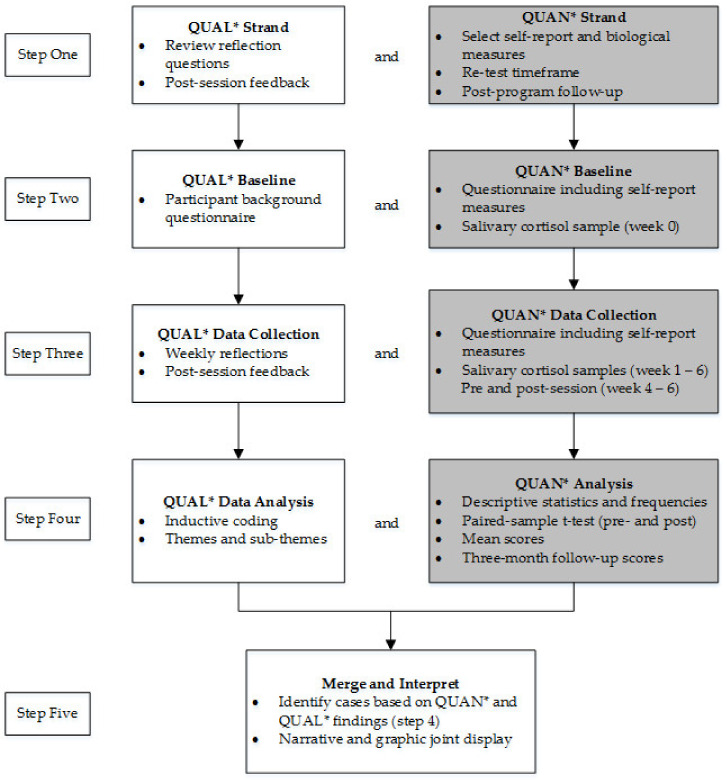
Study design flow chart; QUAL*—qualitative, QUAN*—quantitative.

The small sample size and lack of a control group increased external validity concerns; therefore, a mixed-method design was selected. Greater confidence in the singular conclusion occurs if the different approaches corroborate the findings [46]. Therefore, the inclusion of the qualitative datasets became an essential element to the design. The mixed-method design enabled the causal relationship (mindful attentional awareness, wellbeing, and perceived stress) to be contextualised by the qualitative datasets (e.g., weekly reflections). The quantitative datasets (shaded grey in Figure 1) included in the convergent mixed-methods design reflected the measured utilized in previous research in MBIs and CIs for educators.

In order to provide interpretative rigor in mixed-methods research design [47], the aspect of quality: *interpretive rigor* and the research criterion: *interpretive consistency* was addressed through the process (indicator) of identifying if the inferences are consistent across the datasets and if they closely follow the relevant findings. The criterion: *theoretical consistency* was addressed through the indicator of identifying consistency between the inferences and the theory or existing research in the field [47]. That is, relevant findings from previous research investigating the use of MBIs and CIs for educators were included in the discussion regarding the findings from the present study.

### 1.5. Theoretical Framework for Wellbeing Promotion

**Figure 2:**
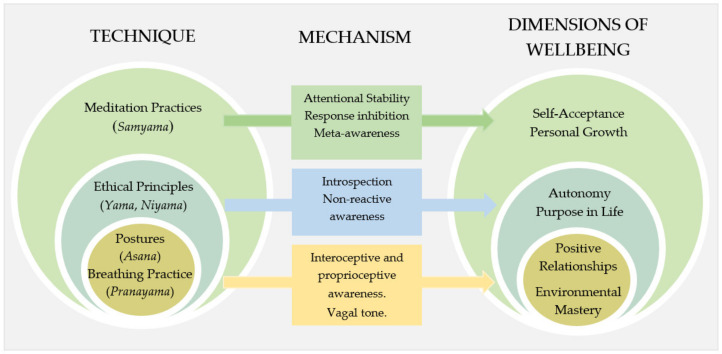
The interaction between the system of yoga and the dimensions of wellbeing.

The proposed model aimed to illustrate the interaction between the dimensions of wellbeing (primarily from the psychological theory of wellbeing [29,48]) and the techniques from an integrated yoga practice (the system of yoga). The model (Figure 2) illustrates the relationship between the techniques, mechanisms, and dimensions of wellbeing. The psychological theory of wellbeing is drawn from the eudamonic approach and includes the dimensions: purpose in life, self-acceptance, environmental mastery, positive relationships, autonomy, and personal growth.

The aim of the present study was not to promote the system of yoga as a panacea for teacher stress. Rather, the system of yoga was included as a set of tools and techniques to assist with stress management and support wellbeing. For a detailed summary of the system of yoga, see [49,50,51,52] (Sanskrit terms are italicised).

The system of yoga includes eight “limbs”. The limbs are viewed as “steps” along a path, and steps one to five are preparatory stages that allow an individual to advance to steps six through eight. In Figure 2, the final stages are grouped as “meditation practices”.

Eight limbs of yoga:

- Breathing practices (Step 1: *Pranayama*)
- Postures (Step 2: *Asana*)
- Ethical principles
  ○ Restraint (Step 3: *Yama*)
  ○ Observances (Step 4: *Niyamas*)
- Meditation
  ○ Sensory withdrawal (Step 5: *Pratyahara*)
  ○ Concentration (Step 6: *Dharana*)
  ○ Meditation (Step 7: *Dhyana*)
  ○ Self-realisation (Step 8: *Samadhi*)

The concept of yoga as a “system” involves more than simple stretches and breathing exercises. Yoga emphasizes how one interacts with others and the world around them. As outlined by [50] (p. 7) “yoga has a number of tools that can help overcome one of the chief factors undermining the health and wellbeing of many in the modern world: an out-of-balance stress-response system.” Yoga and meditation help an individual identify behaviours, perceptions, and dietary changes to promote wellbeing. It is important for an individual to understand the impact of stress and how it manifests [7]. A key argument in the present study is that increasing awareness increases the possibility of creating change.

#### 1.5.1. Techniques

Often the terms yoga and meditation are used interchangeably. However, yoga is an overarching system that includes meditation and mindfulness. Meditation is the process of shifting attention at will onto the object of choice, ideally an undistorted experience. Meditation is operationalized as mental training that develops meta-awareness (self-awareness) and promotes awareness of behaviour (self-regulation) and self-acceptance. There is an emphasis on the shift from self-focused attention and awareness to decentered and prosocial views [53]. Meditation is practiced to cultivate metacognitive awareness through either focused attention (FA) meditation (also called concentrative meditation) or open monitoring (OM) meditation (also called open-focus or mindfulness meditation) [54]. Being mindful and observing the present (mindfulness meditation), focusing attention on the movement and sensations of the body are included in yoga practice [51]. It is necessary to clarify that the practice of yoga is not religious, although the practice originated in Hindu scriptures and ancient Indian philosophies, and a practitioner does not need to subscribe or adhere to a system of beliefs in order to practice yoga. Nor is yoga presented as a religious philosophy in the writings of Patanjali or Buddha; instead, the focus is self-observation [51,52].

Figure 2 does not include “professional development” or “theoretical knowledge” as a technique for promoting wellbeing because the theoretical components are embedded within the overarching techniques and mechanisms. The theoretical components (detailed in [44]) were included in the intervention (detailed below) to provide a deeper understanding of the importance of the techniques and mechanisms for promoting wellbeing and decreasing stress. The theoretical components provided self-knowledge and understanding, which was an important aspect of teacher stress management [6].

The concept of top-down (cognitive) mechanisms influencing and being influenced by bottom-up (physiological) mechanisms [51] are illustrated in Figure 2 as embedded circles. That is not to say that one practice is more influential than the other; rather, it illustrates the bidirectional interaction between the different model components. In the system of yoga, meditation practices are overarching techniques that influence not only cognitive functioning but also emotional and physical responses and reactions; it is therefore illustrated as the largest of the three circles.

#### 1.5.2. Supporting the Dimensions of Wellbeing

The techniques are colour-coded to align with the main dimensions of wellbeing influenced by each technique. Meditation practices primarily align with the wellbeing dimensions of *self-acceptance* and *personal growth*. *Self-acceptance* is a central feature of mental health and optimal functioning. It refers to the attitudes one holds towards oneself [29], for example, “experiencing different aspects of oneself (e.g., one’s past, personality, thoughts and feelings) in a tolerant, receptive and non-judgemental way” [55]. Exercising attentional control is central to action regulation that assists individuals with their ability to re-think habitual appraisals of themselves and others, for example, student misbehaviour [10], which impacts the acute stress response. Moreover, an important aspect of wellbeing is maintaining present-moment attention, engagement, and interest (also referred to as “flow”) [56]. This approach is embedded throughout the system of yoga.

The dimension of wellbeing *personal growth* refers to the ongoing continual development and self-realisation throughout one’s lifespan, which is also referred to as “openness to experience” [28] and which is cultivated through meditation practices. Meditation practices encourage concentration, self-realisation, attention stability, and meta-awareness [50,51,52]. Through meditation practices, there is a shift from self-focused attention and awareness to decentered and prosocial views [53]. It could be argued that this dimension of wellbeing is also promoted through the ethical principles of the system of yoga, for example, how an individiual interacts with the world around them (*Yamas*) and how they treat themselves (*Niyamas*). The overlap between the two techniques is due to the incorporation of ethical principles *into* meditation practices.

The dimensions of *autonomy* and *purpose in life* are primarily cultivated through ethical principles. *Autonomy*, defined by Ryff and Singer [29] (p. 15), is the “regulation of behaviour from within” and an “internal locus of evaluation”. A healthy individual will aim to understand their own values and ideals [57]. This dimension is promoted through the ethical principles (*Yama* and *Niyama*) that focus on ethical, moral, and social aspects of behaviour and self-observation of one’s habits (introspection) utilising non-reactive awareness [50,54]). Contemplative (cognitive) meditation practices are associated with goal setting and the process of self-assessment against the desired goal [49]. *Purpose in life* is a sense of directedness, including goals and intentions [29]. Increasing mindfulness has been reported to provide an individual with the opportunity to shift their focus from chronic stress and self-protection to broader long-term professional development [11].

*Environmental mastery* is a significant dimension of wellbeing and involves “active participation in and mastery of the environment” [29]. The degree of environmental mastery can influence the activities with which an individual chooses to engage. This dimension can be promoted through postures and breathing practices to regulate the stress response; to improve vagal tone, interoceptive and proprioceptive awareness, and consequently, an individual’s ability to respond to threats; and to reduce the impact of chronic stress [58,59,60]. Awareness of bodily sensations can assist in the identification of a situation or behaviour (in oneself or others) that provokes an emotional or physical response. The increase in awareness results in a reduction in reactive and automatic behaviour [12]. In turn, this creates an opportunity for an adjustment in behaviour and perspective, for example, increased awareness of habitual eating patterns or heightened emotional reactions during periods of stress. Physical activity has been associated with the development of health resources such as social relations and care, positive energy, and self-worth [61]. Central concepts to the present study are awareness and perception, both of which are present in an individual’s response to stress and their level of job satisfaction.

Adopting a mindful perspective provides a “mental gap” between stimulus and automatic behaviour; thus, individuals can intentionally regulate their actions in different situations [11] and decrease emotional expression [62]. Consequently, this assists in developing *positive interactions with others*, which is the final dimension of wellbeing. Developing empathy, connection with others, and forming intimate relationships are central to mental health [29]. Establishing positive relationships is a critical aspect of the experiences of ECTs. Guided meditation practices can include ethical principles regarding compassion towards oneself and others [51] and are referred to as integrated yoga practices. Integrated yoga practices may increase compassion and kindness towards others, and guided meditation is reported to increase empathy, forgiveness, and self-compassion [63] as well as to improve classroom interactions [64]. Similarly, increased mindfulness is correlated with self-reported non-reactivity [65] and adjustments in habitual patterns of blame, which are beneficial for teacher wellbeing [11].

Stress can create behavioural changes (e.g., social and lifestyle choices) and psychological and physiological changes that influence an individual’s wellbeing. Consequently, the approach to promoting wellbeing must include strategies to support positive behavioural and physiological changes that reduce the impact of the stress response. Psychosocial and cultural processes provide us with information, shape how we see the world, and influence our bodily responses. This is reflected in the behavioural model of medicine that recognizes the influence of sociocultural and mind–brain processes on an individual’s health [66]. The psychosocial and cultural processes influencing health and, consequently, wellbeing are significant to the present study, and this approach is present in an integrated yoga approach.

## 2. Materials and Methods

### 2.1. Participants

The participant sample included teachers within the early career period (1–5 years of experience). Fifty-one teachers registered for the program, and 19 withdrew before the commencement of the first session due to conflicting commitments (e.g., family events, staff meetings). Thirty-one participants completed the baseline survey and seven withdrew during the intervention or did not complete all the sessions or the participant reflections. The final participant group (*n* = 24) attended all six sessions and completed the required datasets. The demographics of the participant sample were representative of the teaching population as reported by the Queensland College of Teachers Annual Report [67]. The age range was 23 to 58 years (*m* = 36.9, *sd* = 11.7), and 91.7% of participants were female, with 75% in full-time employment and over 60% with industry experience before teaching; additionally, 48.5% were in their first year of teaching.

#### Participant Background

The baseline questions included a combination of open- and closed-ended questions regarding previous experience with yoga or meditation and existing exercise routines, providing relevant background information and creating a context for the results. The relaxation activities were inductively coded [68] as social, physical, personal, and ambiguous (Table 1). Ambiguous activities could not be classified as social (group) or individual activities. The responses for the combined open-ended and closed-ended questions (exercise routine, previous experience with yoga or meditation, and wellbeing initiatives in the workplace) are reported as a narrative discussion below [68].

Of the 24 participants, 58% (*n* = 14) said they had previously attended a beginner’s yoga or meditation class (e.g., a one-off class at a gym). Two participants completed regular yoga or meditation classes, and 33% (*n* = 8) had no previous experience with either yoga or meditation. Of the 66% (*n* = 16) of the participants that completed regular exercise, 50% (*n* = 8) attended weekly or daily gym and personal training sessions. The remaining participants were involved in team sports (e.g., netball), walking (daily), martial arts classes, swimming, and bicycle riding. Four participants felt that doing exercise was a form of relaxation activity. One participant explained that they wished they had more time for relaxation activities. Thirty-three percent (*n* = 8) did not have an exercise routine or complete any physical exercise.

The participants indicated (combination question) if they were aware of any wellbeing activities available at their school (e.g., fitness classes, staff morning tea, or wellbeing professional development). Of the participants, 45% (*n* = 11) indicated there were no activities or initiatives supported by their school, 33% (*n* = 8) were unaware of any activities or initiatives, and 20% (*n* = 5) said they attended the wellbeing activities or initiatives (e.g., professional development, staff birthdays, afternoon tea, social activities, and positive notes).

### 2.2. Procedure

Approvals were received from the Human Research Ethics Committee from the University of Queensland, the Department of Education and Training Queensland and Brisbane Catholic Education. The Code for Responsible Conduct of Research from the National Statement on Ethical Conduct in Human Research (2007) [69] was adhered to throughout the study.

The project details were distributed to 49 schools (Prep–Grade 12) in Brisbane, Queensland, Australia. The project information letter detailed the requirements of the study, and the self-selected participants (*n =* 51) registered for the program. An information session was held one week prior to the start of the program to provide detailed instruction regarding data-collection methods. The participants completed the baseline survey including informed consent and received a cortisol sample collection kit. The surveys formatted in Qualtrics [70] were distributed by email (weekly and three-month post-program). To reduce sampling errors, the researcher confirmed the data collection procedures each week.

The sessions were held on the same day of the week at the same time outside of school hours (4:00 p.m.–6:00 p.m.) at a central location for six consecutive weeks in the second semester of the academic year. The participants received take-home materials and pre-recorded physical practice sessions (e.g., yoga sequences and guided meditation).

Cortisol sample kits were collected by the lead researcher each week. The samples were collected the day after the session. The cortisol awakening rise (CAR) sample was collected within 30 min of waking, and the evening (resting) sample was collected within 30 min of going to bed. The instructions for collecting the samples provided in the IPRO Cube Reader Manual were summarized and explained to the participants. The oral fluid collector (OFC) swab collected 5 mL of fluid and included a volume adequacy indicator (colour change) [71]. The weekly reflection and post-session feedback survey was emailed to the participants. The participants were required to create participant codes to ensure the anonymity of the data collected. The participant codes were used to track the participant responses.

### 2.3. Intervention

The Integrated Wellbeing and Stress Management program (intervention) included three distinct but related topics: psychological wellbeing (weeks 1 and 2), physiological wellbeing (weeks 3 and 4), and social (interpersonal) wellbeing (weeks 5 and 6). The Integrated Wellbeing and Stress Management program was piloted with pre-service teachers as part of a Doctoral research project (see, [72]). The findings from the pilot (participant experience) indicated the importance of the physical component; therefore, the intervention was reviewed and extended (six weeks) to allow for the inclusion of physical practice and to provide an opportunity for the participants to practice the techniques. The facilitator and program developer had over seven years of experience as a yoga and mindfulness teacher trainer with national and International Yoga Teacher Registration (i.e., Yoga Australia–Level 2 registration and Yoga Alliance–Level 1). The 12-h manualized program included theoretical and practical components. The participants were provided with necessary equipment (e.g., yoga matt, blanket, eye-pillow). Each weekly 2-h session included the same format and the physical practice and exercises increased from week one to week three and included a 60-min physical practice in weeks 4, 5, and 6. The PowerPoint slides were scripted, and the physical practice had the same method of delivery and instruction to ensure the consistency of session delivery. A detailed outline of the intervention has been reported elsewhere (see [44,72]).

Session structure:

- 30–45 min of theory to support the activities for the session
- 15 min of reflection and group discussion activities
- 30–60 min of movement/posture practice
- Home practice was determined by the participants

Theoretical component:

Psychological wellbeing (weeks 1–2)

1. The impact of stress
2. The benefits of relaxation
  Physiological wellbeing (weeks 3–4)
3. The importance of exercise
4. Diet and stress

Social (interpersonal) wellbeing (weeks 5–6)

5. Self-compassion
6. Professional relationships and isolation

Physical component:

- Instant relaxation exercises
- Guided meditation—e.g., Yoga Nidra, body scan meditation
- Supported restorative postures
- Yoga sequences—e.g., Sun salutations, supine and seated postures.

### 2.4. Measures

#### 2.4.1. Qualitative Datasets

The reflection questions were specifically designed for the present study. A phenomenological approach was selected to understand the experience of the participants in the program (i.e., to understand people’s everyday experiences) [73]. The post-session feedback included a description of the intervention (open-ended questioning), for example, *what was your experience of the session*? and open-ended and closed-ended questioning, for example, *do you feel you could apply the program content or activities personally or professionally, provide an example*? The weekly reflection questions identified benefits and disadvantages experienced (subjective questions) and significant events and were a combination of open-ended and closed-ended questions [74]. For example, *has anything happened this week that has had an impact on how you have been feeling (e.g., work/family/study/**positive or negative events** that may have influenced how you have been feeling)?*

#### 2.4.2. Quantitative Datasets

The pre- and post-program self-report measures included the Perceived Stress Scale (PSS; [75]), the Mindful Attention Awareness Scale (MAAS; [76]), and the Personal Wellbeing Index (PWI; [77]). The PSS is an established measure for psychological distress and includes 10 items (5-point Likert scale). The coefficient alphas ranged from 0.77–0.78 [62], and the reported norm was 13.7 [78] in a US population sample (*n* = 2387). Scores of 20 and above were classified by the authors as “high stress”.

The MAAS is a 15-item 6-point Likert scale that was originally developed to assess the link between mindfulness and wellbeing [76]. The MAAS is more indicative of naturally occurring (dispositional) mindfulness, that is, mindfulness as a state, not a trait. Previous MBIs have investigated mindfulness as a trait and that was not the aim in the present study [44]. The MAAS has high convergent validity, test-retest reliability, and coefficient alphas ranging from 0.80–0.90, and the reported norm for adults was 4.2 (*n* = 436) [76].

The PWI was developed from the Comprehensive Quality of Life Scale (ComQOL) [79]. The eight items are scored on an 11-point end-defined response scale. The eight quality of life domains are represented including: safety, community-connectedness, future security, achievement in life, standard of living, health, relationships, and religion/spirituality. The PWI was scored to produce an overall score for subjective wellbeing. The coefficient alphas ranged from 0.70–0.85, and the reported norm in Australia was 73.4–76.4 points [77].

The morning (CAR) and evening (resting) levels were included as a biological measure for the physiological stress response. The samples were collected the day after the program session for the duration of the intervention (baseline to week 6). Pre- and post-session samples were collected for weeks 4–6 to determine the immediate response to the 60-min physical practice. The negative feedback action of cortisol regulates the hypothalamus-pituitary-adrenal (HPA) axis, via the cortisol receptors in the brain (e.g., hippocampus, hypothalamus, and the pituitary gland). This process suppresses the secretion of corticotropin-releasing hormone (CRH) and adrenocorticotropin (ACTH) [80]. CRH is secreted from the hypothalamus (paraventricular nucleus), which triggers the neuroendocrine stress response from the HPA-axis. This endocrine pathway stimulates the secretion of catecholamines from the adrenal glands (for example, epinephrine and norepinephrine). The main drivers in the stress response are the sympathetic nervous system and epinephrine; the role of cortisol is to regulate the response [66].

### 2.5. Data Analysis

The research questions were drawn from an explanatory mixed-methods content-focused approach [81]; therefore, the qualitative data were used to provide a context to the quantitative findings. The steps for conducting inductive coding by Creswell [68] were followed. After the first sweep, the text data was divided into segments (unitization), and the segments were coded (margin coding). The domains were identified, and a subsequent sweep of the data was completed, followed by grouping codes in each domain, refining codes into themes and refining themes [82]. Each domain (reflection question) had a narrow focus; therefore, there was not a broad range of themes. Cross-case (participant) analysis was completed for each domain, and the themes and sub-themes were identified.

Combination questions (open- and closed-ended) were reported as a narrative discussion, and graphic displays were included to illustrate the closed-ended questions [68]. The participants’ experiences are conveyed in direct quotes throughout the results [83] to strengthen the face validity of the research [84].

A sequential design was implemented to identify the cases [81] that were subsequently presented as a narrative discussion. The sequential design involved:

1. Reviewing all the individual data included in the inductive thematic analysis.
2. Cases were selected to represent the demographics of the teaching population.
3. Cases selected reflected the overall trend of the participant sample, for example, changes in salivary cortisol levels, perceived stress scale, and attention awareness.
4. Complete datasets for the self-report, biological measures, and qualitative reports (e.g., weekly reflections, session feedback, and post-program follow-up) were required to represent the wholeness of the participant experience.

The self-report measure scores (PSS, MAAS, PWI) were reported for each individual case study. The salivary cortisol levels for the individual case studies were reported in comparison to the means scores of the whole participant sample.

## 3. Results

Multiple perspectives and quotes from the original data are reported for each section [68] and pseudonyms were used to maintain anonymity of the participants.

### 3.1. Reported Benefits and Disadvantages

Two participants reported a disadvantage from participation in the program relating to attending sessions after school hours, travelling to the location, and adjusting planning, preparation, and personal time. For example, Anthony described a disadvantage as “having to adapt my personal time management as getting home after the program at around 7:00 p.m.” (week 4).

**Figure 3:**
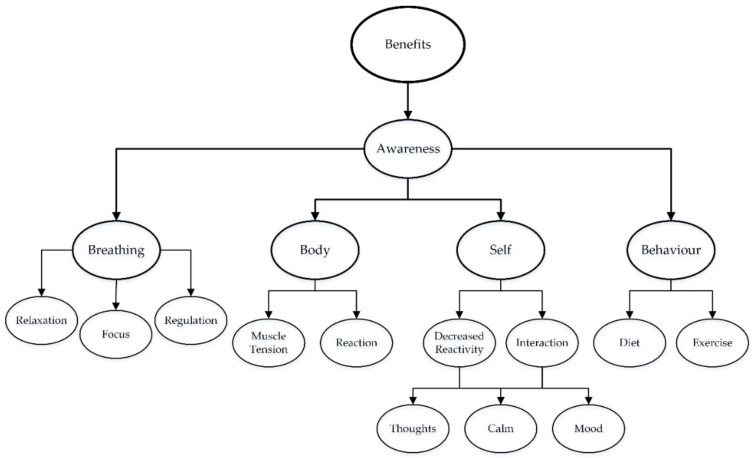
Coding of themes for the domain-reported benefits.

The participants consistently reported benefits across the duration of the program. The theme of awareness included sub-themes related to breathing, body, the self, and behaviour (Figure 3).

The increased sense of awareness of the Self included decreased reactivity and interaction with others, an increased sense of calm and awareness of thoughts, and improved sleep quality. For example, Mary said “I’ve felt lighter, happier” (week 2) and “I feel like I have been calmer and not getting frustrated as easily” (week 3). Similarly, Jo shared that she had been “feeling happier with family, partner, school students” (week 6). Whereas Anthony indicated a benefit in a professional context when he explained, “I’m not stressed doing my exams, marking and report writing” (week 3); his response suggested increased awareness of the stress response. Isla shared that she had “awareness of needs, awareness that needs are not being met, awareness of taking time to see and find positive things” (week 4).

Penny highlighted the link between increased awareness and changes in behaviour and implementing techniques. For instance, in week 5, she shared that she felt “more mindful of trying relaxation and taking time to stop for lunch.” However, Lesley reported she had “more awareness of thoughts and emotions. Ability to use techniques (yoga) to support finding a balance” (week 2). Behaviour changes included diet and healthy choices. For instance, Rachel described a change to meal preparation: “I ordered some pre-cooked meals so I would have a proper lunch every day and save time cooking. I was aware of how some foods made me feel” (week 5).

The reported changes in awareness related to reactivity and body sensations (proprioception and interoception). For example, Leonie shared, “I feel calmer and less heightened, I focus on breathing deeper a few extra times a day” (week 5), suggesting breathing may have assisted with regulation (i.e., physical or emotional stress response). Examples of increased body awareness and applying breathing techniques to calm and reduce the stress response, feelings of anger, and increased focus are provided in Table 2. Decreased rumination was suggested by Ashley’s response: “I find it easier to relax and meditating has helped me to let go of the things that hang on after work” (week 6). Noticing changes after applying the techniques and strategies was shared by Fiona:

More awareness of areas of my life that I need to change to allow my body to rest and recover. Bought a fitness watch and noticed that it was measuring my stress levels as quite high the whole time I’m asleep. The night I did some breathing exercises straight before going to sleep, my stress score stayed lower for the whole night (week 3).

The reported benefits suggested a change over time. For example, in week 2, Tanya explained that she was “being more mindful of my thoughts” and by the final session (week 6) “I feel more in control of my thoughts, feelings.” This suggested an increase in awareness, which may have provided an increased sense of control and decreased habitual or reactive behaviour. Similarly, Ellen reported “being aware of the need to not overstress. Conscious efforts to ensure this” (week 3) and “becoming more aware of the importance of my wellbeing, easy, quick ways I can include this into my life and also my daily classroom” (week 6). Table 2 provides a comparison of the difference in the responses. The focus shifted from noticing or identifying behaviours or sensations to implementing strategies and making changes. The increased sense of awareness relates to the mechanisms included in the system of yoga (listed in Figure 2).

In the final weekly reflection (week 6), eleven participants (45%) had not considered or included the techniques into their classroom routine or with individual students, and eleven participants (45%) had included or considered using the techniques (no response, *n* = 2). For instance, Cathy explained that she had introduced mindful breathing and yoga to her classes and noticed the students were “less restless.” Additionally, Isla said she had included “mindfulness activities, breathing, noticing muscle tightness and relaxing. With individual students and small groups.” Similarly, Tanya introduced body scan meditation and deep breathing to her classes.

In the three-month post-program survey (*n* = 17), no disadvantages were reported. Seven participants reported unexpected benefits suggesting changes in behaviour. For example, Leonie shared: “more awareness and generally being more measured and mindful in my approach to my wellbeing” and Janet reflected “incorporating some of the practices taught into everyday life”.

### 3.2. Program Content and Application–Participant Experience

**Figure 4:**
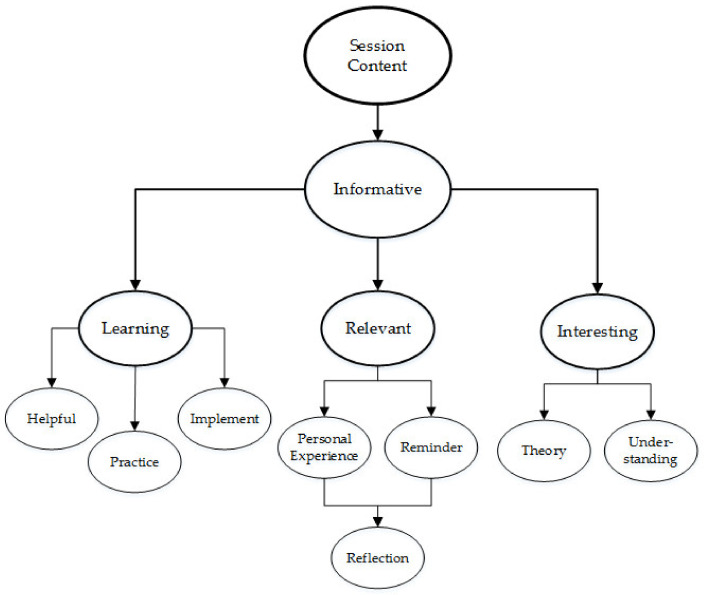
Graphic summary of the themes and sub-themes from the participant post-session feedback (session content).

The program was described as informative and provided an opportunity to learn new information, which was helpful and relevant to their personal experience. The program was described as practical and provided the opportunity to practice (experience) the techniques. A summary of the content description is illustrated in Figure 4. The theoretical component provided a greater understanding of the theories and concepts underlying the techniques. For example, Beth described Session One as “interesting to learn more about mindfulness, how to recognise my own stress and holistic strategies to care for my mental wellbeing”. Fiona explained “I expected to be given some strategies to relax and felt that I was given quite a few ideas that I could actually put into practice. I particularly enjoyed the scientific background, helped me to understand the strategies better”.

The opportunity for discussion with peers and colleagues was highlighted by Ashley: “The session was informative, and I enjoyed the discussion between colleagues regarding teacher challenges and teacher triumphs” (Session Six). In addition, the participants reflected that the content served as a reminder of what they should be doing to prioritise their wellbeing. Jess described Session One and highlighted that learning information had practical applications:

It exceeded my expectations. The theory and literature behind it was fascinating and I love how it was linked back to how you can use it in the classroom as well as still making it about the educator.

Finally, Mary shared a personal reflection: “I love learning about ways to improve my mood and how I’m feeling as I can easily become angry and frustrated with my family” (Session Three).

**Figure 5:**
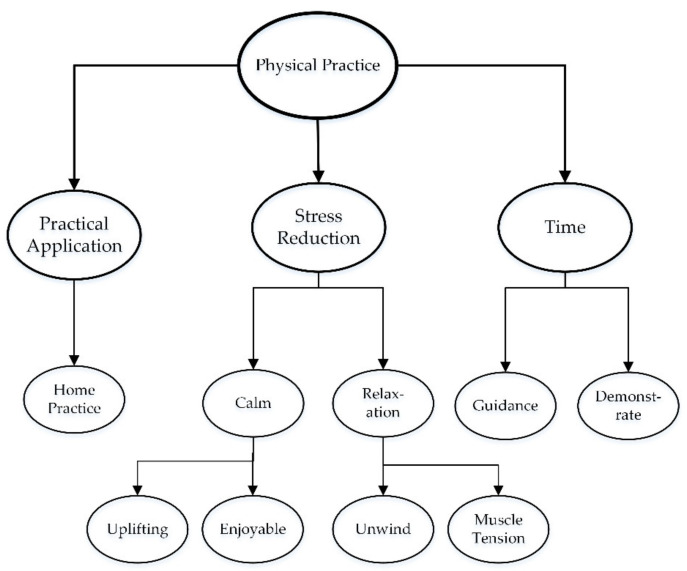
Graphic summary of the themes and sub-themes from the participant post-session feedback (session description).

In contrast to the findings from the pilot study [72], the session descriptions highlighted the physical practice, suggesting that the practical component provided the opportunity for stress reduction and time to experience (and learn) the techniques (Figure 5). For example, Kelly explained:

I really enjoyed learning the theory behind yoga. I am struggling with back and shoulder pain at the moment, so I found the Body Scan meditation great as it helps me to focus on other parts of my body not the pain. I feel this really helps me to relax. Again, I enjoyed finishing the session with the practical side of the session. I leave feeling relaxed (Session Two).

The physical practice provided the participants with stress reduction, guidance, and the opportunity to reflect on how they could apply the techniques (practical application). As suggested by Mary’s description of Session Three: “very enjoyable, always feel very relaxed when finished and excited to utilise the strategies and learn more information” and Session Six: “I always learn something new, like how important mindfulness is and being aware of thoughts and how they affect feelings”.

Stress reduction included increased relaxation, creating a sense of calm. The participants explained that the sessions provided the chance to unwind and increased relaxation. Isla explained, “I leave feeling calm and relaxed,” whereas Anthony said, “the yoga stretching is effective for de-stressing after the school day” (Session Six). Ellen shared: “I enjoyed the exercises as they are practical and something which I believe I will enjoy and benefit from” (Session Six). In addition, the practical component (physical practice) provided time to practice the techniques and clear guidance and instruction. For example, Sonya said, “doing the exercises repeatedly with clear instructions helps me remember them and do them correctly at home” (Session Five). The importance of having time was explained by Tammy “after a frustrating week it was nice to spend some time on me” (week 5).

The session descriptions included potential practical application of the program content. For example, integrating the techniques into home routines (personal application) was summarised by Kelly:

I really like how we end each session with yoga and meditation as it helps refresh my body and mind for the week. I also feel like I am more successful in relaxing my body when I do the yoga and meditation sessions at home (Session Three).

### 3.3. Program Content Application–Personal and Professional Contexts

**Figure 6:**
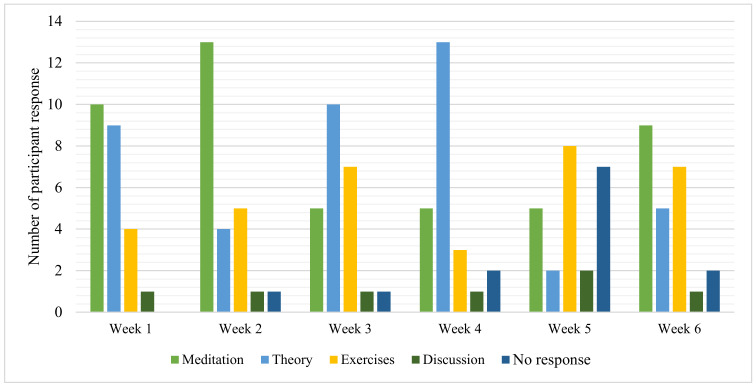
Useful session activities (*n* = 24).

Each session included the same format and activities, however the practical component increased in length across the duration of the program, and the theoretical component subsequently decreased. For example, Session 1 included 60 min of theory and 30 min of physical practice, whereas Session 6 included 30 min of theory and 60 min of physical practice. Participants identified the activities they found useful, and the activities were listed as: “meditation,” “theory,” “exercises,” and “discussion” (Figure 6).

Discussion activities were the lowest across all six weeks, whereas meditation and theoretical activities were consistently reported as useful. For example, Jess explained:

The theory component helps explain how my body and brain works and gives me the information I need to decide what will work for me. I found the yoga and meditation session really helpful (Session Two).

Over 65% of the participants consistently reported applying the content to both personal and professional contexts. For example, after Session One, 75% of the participants (*n* = 18) felt they could apply the content professionally and personally, whereas four participants felt they could apply the content personally only. In the reflection from the final session, nineteen participants provided an example of how they would apply the content in the classroom *and* personal situations. Examples included teaching breathing techniques and strategies to their students or small groups (behaviour management strategies) and promoting wellbeing more generally across the school with classes and colleagues. For example, Ellen explained: “I want to introduce a wellbeing program in my classroom next year”, whereas Anthony shared: “personally after school at home to unwind after a tough day” and Penny reported: “personally, get back to yoga and daily practice. Professionally, use some of the relaxation content with high school students who I meet with weekly”. Finally, Tammy explained:

Personally: I will make goals to implement some of the activities into my daily schedule. I will also include my children. Professionally: I have ideas of making mindfulness a daily activity in my class next year. Personally: I want to start to a gratitude journal to help me change my perception.

### 3.4. Participant Reflections and Self-Report Measures

The contextual information pertaining to the participants’ experiences may relate to the self-report and biological measures changes. A combination question (open- and closed-ended) was included to identify an influential event from the previous week that may have had an impact on how the participants were feeling. The participants indicated whether the event was “positive” or “negative” or “neutral” (Table 3) and provided a brief description. Week 1 referred to the week before the start of the program. The event description data were collated for the duration of the program and represented in a joint display for negative events (*n* = 51) (Table 4) and positive events (*n* = 31) (Table 5).

The open-ended question resulted in multiple responses from the participants, including both negative and positive examples. For example, Ellen shared: “negative: student behaviour and parent issues. Positives: super-engaged students, secured a job interview.” Of the total “negative” events, 65% (*n* = 34) were work-related and 30% were student behaviour concerns.

The “positive” events that related to work typically focused on feedback from colleagues, parents, and students regarding lesson activities and student achievement. The “positive” events were typically related to family and personal circumstances, for example, the availability of time and appreciation for spending time with friends and family. Identification of a positive or negative event was intended to create contextual information for merging the datasets. An exploration of the experiences of the participants below provides contextual information surrounding the qualitative data.

### 3.5. Case Studies

#### 3.5.1. Ashley–First-Year Teacher

The self-report (PSS, MAAS, PWI) and biological measures for Ashley represented the overall trend from the participant sample (participant sample findings are reported in [44]). Ashley was a first-year upper primary teacher in fulltime employment (aged 25–29 years of age) without any dependents. She did not have any previous experience with yoga or meditation and shared that she “had a gym membership but is struggling to find time to use it”. She described her work–life balance prior to the program as “chaotic and unbalanced”. Her motivation for joining the program was “learning to slow down and schedule time for my wellbeing”.

**Figure 7:**
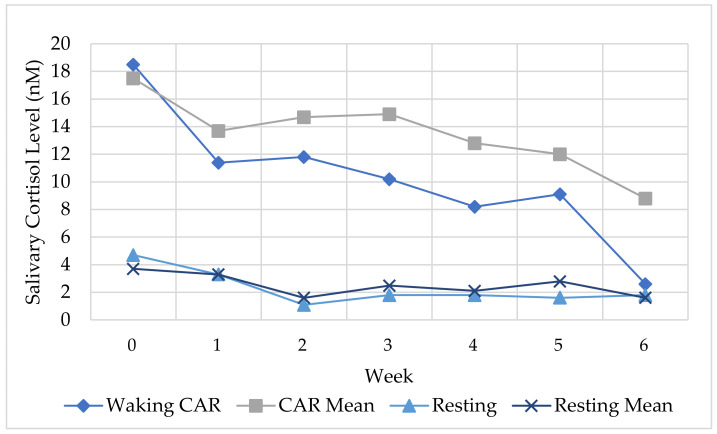
Change in waking (CAR) and resting salivary cortisol levels from week 0 to week 6 for Ashley.

The results indicated a decrease in waking (CAR) (−15.9) and resting (−2.9) salivary cortisol levels from the baseline (week 0) to week 6. Ashley’s weekly levels are illustrated in comparison to the participant sample mean in Figure 7.

Ashley’s pre-program PSS score of 19 was above the age-matched reported norm population score (12.3–13. 7) [78] and decreased to 16 post-program (Table 6). Ashley’s MAAS score of 2.93 (pre-program) was below the reported norm score (4.2) [76] and increased to 4.13 (post-program). The group mean scores have been included in Table 6 (detailed datasets from the full participant group are reported in [44]).

Ashley’s reported benefits suggested a subjective increase in awareness (interoceptive and proprioceptive awareness), a recognition of feelings and sensations, a sense of calm, and decreased muscle tension supporting a change in perceived stress and attention awareness. For instance, in week 2, she shared that she felt “calmer and more aware of when I am starting to feel heightened” and in week 3 she shared: “methods to assist in calming. In week 4, she shared that she was “finding it easier to calm myself and using the restorative pose a lot”, and, by week 6: “I find it easier to calm myself because I can recognise the feelings and sensations more easily”.

Ashley highlighted the importance of allocating time in her schedule when she shared: “it was great to spend allotted time relaxing and taking time to think about your day and week and body” (week 6). Ashley’s subjective wellbeing (PWI) score of 43 was below the norm mean range (70–80) [77], which increased to 59 (post-program), indicating an increase in subjective wellbeing. Ashley’s description of the program highlighted the importance of integrated (holistic) wellbeing:

Very informative and I liked how the presenter talked through all of the science behind it and how there needs to be a holistic view encompassing stress management, social engagement, and physical health.

The three-month post-program follow-up indicated that the change in PSS, MAAS, and PWI was maintained. Ashley said she had started classes at the gym and online meditation, indicating a change in behaviour. She made the connection between teacher functionality and wellbeing when asked if teachers needed to be provided with strategies to promote wellbeing, explaining: “you need to be well to do this job and sometimes we give everything to the kids and leave no time for ourselves”.

#### 3.5.2. Case Study Two: Sarah–Third-Year Teacher

Similarly, Sarah’s experience in the program reflected the overall trend from the participant sample [44]. Sarah was a third-year lower primary teacher in fulltime employment. Aged between 45–49 years, she had two dependents between eight and ten years of age. She had previous industry experience in administration and information technology support. Sarah had limited previous experience with yoga (e.g., she attended one–two classes), did not complete any fitness activities, and used walking or watching a movie for relaxation. She described her work–life balance prior to the program as “not good”. Her motivation for joining the program was to “start looking after myself and be happy. Be happy at home and work”.

**Figure 8:**
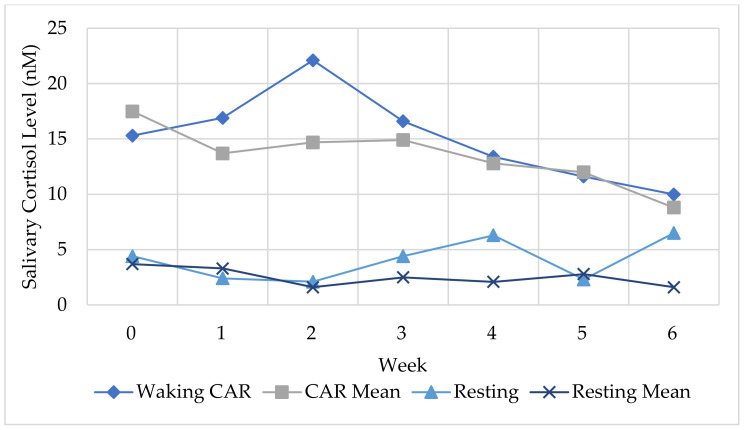
Change in waking (CAR) and resting salivary cortisol levels from week 0 to week 6 for Sarah.

Sarah’s results included a decrease in waking (−11.6) and resting (−0.08) salivary cortisol levels from the baseline (week 0) to week 6 (Figure 8). Sarah’s changes in CAR may be reflected in her consistent application of the techniques across the duration of the program.

Sarah had a pre-program perceived stress score of 24, which is above the “high stress” score of 20 [78], which decreased to 12 (post-program) (Table 7). Her MAAS score of 2.8 was below the reported norm scores (4.2) [76] and increased to 4.2 (post-program). Sarah’s reported benefits indicating increased awareness of behaviours, thoughts, and self-talk (interoceptive and proprioceptive awareness), which changed over the duration of the program. After the first session, Sarah reported she was “thinking a lot more of my sleep and eating patterns. Trying to walk more. Trying to take 10 min each night to listen to relaxing music”. By the third session, she was “feeling more positive” and using breathing and stretching techniques and “trying to get to bed before midnight”.

Her PWI score of 46 was below the norm mean range and increased to 68 (post-pro-gram). The three-month post-program follow-up suggested that the changes in PSS, MAAS, and PWI were maintained. Sarah’s final session description suggested an increase in awareness (relating to autonomy): “I have learnt extremely valuable information in every session. I am feeling happier than I have in 10 years. Feeling mentally and physically more aware and stronger”. Similarly, in the final (week 6), she shared “feeling positive more than not. Six-week program a big plus to get me into a good mental state with meditation and implement a solid physical routine”. In addition to feeling physiological benefits, Sarah reported she was “feeling more positive in every area of my life” and she could “turn negative thinking around”. Her decrease in PSS and increase in PWI may be reflected by her increased ability to identify patterns in behaviour (e.g., negative thinking or ruminating) and decreased negative reappraisal of situations.

Sarah described the program content (post-session description) as providing interesting, relevant information that was applicable to her life. She explained the first session “made me understand the importance of a balanced lifestyle. Time for me if OK. Exercise and a healthy eating routine are needed to stay at our best and give our best”, therefore suggesting an awareness of the importance of wellbeing and functionality.

At the three-month post-program follow-up questionnaire, Sarah said she had started a fitness routine, which included four exercise sessions a week, yoga, and meditation three times a week. Her reported benefits three-months post-program were “happiness and feeling good about myself. Handling home and work situations more calmly. Ensuring I take time for myself”.

The participant experiences reported in the weekly reflection and post-session feedback responses indicated a change in awareness surrounding the self, bodily sensations (e.g., breathing and reactions), behaviours, and thoughts. The Integrated Wellbeing and Stress Management program provided an opportunity to learn new information and practice the physical techniques; in addition, the participants highlighted the importance of having time to reflect, engage with peers, and take time for themselves. The participants identified the program content that was applicable in professional and personal contexts.

## 4. Discussion

### 4.1. Benefits and Disadvantages Experienced

Teachers suffer from role overload and a variety of time pressures. Therefore, offering a professional development program outside of school contact hours presented limitations for participant attendance. As indicated in the results, only two participants expressed a disadvantage from participation in the program relating to time pressure. The sessions were held after school (4:00 p.m.) and required travelling to a central location; consequently, the participants needed to adjust their schedule. Due to the program being conducted as a research project and not in conjunction with school-supported professional development (e.g., sessions held within school hours or allocated staff meetings), the timing of the sessions could not be avoided.

Over 93% of the participants reported a benefit from participation in the program (weeks 2 to 6). The benefits reported echoed the pilot study [72] with relation to an increased sense of awareness. However, the program in the present study occurred over a longer period and included a longer physical component in each session, and the large dataset provided a detailed understanding of the benefits experienced.

The increased sense of awareness related to a sense of self, such as noticing moods, interactions with others, thoughts, reactivity, and attempting to regulate emotions and increased sensitivity to thoughts and emotions, has been previously reported by teachers in MBIs when reappraising situations [85]. It is suggested that the increased sense of awareness decreases habitual reactive tendencies [50], and it is proposed in the present study that increased sense of awareness supports the dimension of wellbeing: *positive interactions with others* [29]. Vagal stimulation results in an increase in the promotion of prosocial hormones (e.g., oxytocin). Through increased vagal tone, prosocial hormones create positive emotions (e.g., empathy, affection) [58]. The findings in the present study reflect previous research investigating a 10-week MBI for educators [86] where the participants reported an increased awareness of the reactions of the self and others. Likewise, teachers have reported experiencing an increased sense of compassion and kindness towards students creating conflict in the classroom [86,87].

The reported benefits suggested an increased sense of awareness of thoughts and rumination. The ability to re-think appraisals of situations (and the self) has implications in professional contexts, for example, student misbehaviour [11]. It is proposed that meditation practices support the dimensions of wellbeing *self-acceptance* and *personal growth* [29]. Through meditation practices, ethical principles (awareness of self and others) can be included, and attentional stability is created. Maintaining attentional stability (present in meditation practices, *Samyama*, Figure 2) can reduce negative reappraisal and rumination and contribute to cognitive factors (e.g., response inhibition) [49,50].

The benefits reported included decreased reactivity and an increased sense of calm, which supported the physiological benefits of implementing the techniques and increased prosocial emotions. Previous research has highlighted changes experienced by participants in MBIs for educators relating to education experience, dealing with conflict, anxiety, and changes in the classroom [88] and using the techniques to create a feeling of calm before dealing with a challenging classroom situation [87]. From a physiological perspective, changes in mood have been previously linked to GABA levels in research investigating the difference in benefits experienced by participants in the general exercise and Hatha yoga programs [89].

An increased awareness of breathing and implementing breathing practices was shared by the participants. Diaphragmatic breathing promotes vagal stimulation, resulting in a decrease in reactivity or overactivity of the amygdala, and can result in rapid changes in attention, behaviour, emotion regulation, and perception [90]. The participant reflections highlighted the rapid changes that can occur (outlined in the results) when implementing different breathing practices. A reported benefit of breathing practices for teachers participating in an MBI included the ability to ground through awareness of breathing and deal with conflict in school settings [88]. Breathing practices stimulate the parasympathetic nervous system via the vagal network, which downregulates the stress response (physical and psychological) and assists the practitioner in calming the body and mind, increasing mind–body awareness [49]. Breathing practices are also included in the practice of different postures. The dimension of wellbeing that could relate to breathing practices and postures is *environmental mastery*, which refers to how an individual participates in the surrounding environment [29]. Responding to the environment or a situation can be influenced by improved vagal tone, interoceptive and proprioceptive awareness, threat detection, and adjustment of behaviour that results from increased awareness [11,12]. Awareness of breathing closely aligns with and supports the development of body awareness. The reported benefits included improved sleep quality as a result of incorporating breathing techniques into daily routines.

Experiences that cultivate bidirectional mechanisms create reciprocal feedback and, consequently, have a therapeutic effect on the stress response, such as reductions in anxiety and anger and an increased ability to relax and process stressful situations [91]. This was reflected in the benefits shared by the participants, for instance, increased awareness of the physical and emotional stress response, muscle tension, and reactive behaviour. The findings reflect the experiences of participants in the Cultivating Awareness and Resilience in Education (CARE) program [87] that indicated an increase in awareness of holding tension in the body and of how to release it. Awareness of body sensations can assist in the identification of a situation or behaviour (in oneself or others) that provokes an emotional or physical response. The increase in awareness results in a reduction in reactive and automatic behaviour [12].

The dimension of wellbeing *environmental mastery* could also be linked to ethical principles (Figure 2) surrounding behaviour in the system of yoga, which was evidenced in the reported benefits relating to changes in behaviour. The participants adjusted their behaviour and the activities they engage in (*environmental mastery*) and considered their degree of self-care (ethical principles) and consequently made changes to their diet and exercise routines. Similarly, the reported findings from the CARE program [87] included greater self-awareness and increased physical and emotional health.

### 4.2. Program Content and Application

The post-session feedback reflected the findings from the pilot study, highlighting the importance of scientific evidence and understanding the underlying theories behind the techniques. The participants also felt the sessions provided an opportunity for stress reduction, practical application, and creating time for rest and relaxation. Previous research has indicated that teachers attending MBI programs reported feeling calm, relaxed, and centered [85,86] in addition to experiencing a decrease in the stress response [87,88]. Healthy individuals are said to aim to understand their own values and behaviours [57]. The application for the content in personal contexts related to *autonomy* (dimension of wellbeing), for example, the ability to adjust and regulate behaviour [29].

The applications of the program content in professional contexts reflected the changed teaching practices reported by participants in MBIs for educators, such as teachers adapting mindfulness-based strategies for the classroom to assist students with decreasing muscle tension and increasing the sense of calm in the classroom [85,88]. The participants indicated that they felt their students were less restless as a result of including the strategies (e.g., breathing techniques and gentle stretches) into their class routine, and they had selected a time of the school day to incorporate the techniques (e.g., after lunch), highlighting a change in classroom management and planning [88]. The reported benefits included using the techniques (e.g., breathing) to calm individual students after a conflict or during a challenging classroom incident. In addition to providing examples of techniques to use in the classroom for releasing tension (e.g., breathing techniques throughout the school day), the participants reported broader intentions, such as, plans to implement a wellbeing program or initiative in the school and to teach students and colleagues about the underlying principles for the techniques. Increased sense of directedness, goal setting, and intention relate to the dimension of wellbeing: *purpose in life* [29].

### 4.3. Intervention Results and Participant Reflections

The aim of Research Question 2 was to determine how the participant reflections (experiences) helped to explain the quantitative results. The aspect of quality in mixed-methods research: *interpretive rigor* and the research criterion: *integrative efficacy* have been addressed through the process (indicator) of merging the inferences from each strand (qualitative and quantitative datasets) to form meta-inferences [47] (p. 309). Therefore, the discussion will provide contextual information (participant background and reflections) and quantitative findings (self-report and biological measures) to present the meta-inferences relating to the dimensions of wellbeing.

The literature surrounding stressors for teachers includes classroom factors such as student motivation, misbehaviour, and differentiation in conjunction with school-based factors such as decreased autonomy and control over curriculum and policy and increased workload and accountability measures [15,62,92]. The reported events (“positive” and “negative”) reflected the research surrounding professional identity formation—for instance, ECTs being exposed to contradictory attitudes to teaching and learning (colleagues), maintaining emotional distance from students, and appropriately managing student behaviour [41] and avoiding over-exertion due to workload (not knowing when enough is enough) [40]. The demanding work conditions of the profession are magnified for ECTs due to inconsistencies in support, mentoring, and accountability measures (e.g., performance assessment) [15,30]. In the present study, the significant events reported throughout the program presented in the results (Table 4 and Table 5) echoed the literature surrounding ECT stress and wellbeing. For example, 65% of the “negative” events related to professional contexts. In particular, 30% of the total “negative” events related to student behaviour, followed by interactions with colleagues and workload.

Psychological wellbeing relies on positive social relationships [29], and the “positive” events in the present study typically referred to social relationships (e.g., professional and personal). The “positive” events were more evenly distributed across personal and professional contexts, and, in addition to spending time with family and friends, having time for personal reflection and relaxation was emphasised. The importance of collegial support and feedback (e.g., students and parents) was highlighted; for example, 25% of the total “positive” events related to performance review, observations, and receiving positive feedback from colleagues, students, and parents. For ECTs, the process of connecting with colleagues, discussing and reflecting on events, is vital for professional identity formation and coping [9] and for reducing feelings of isolation [36,37]. One of the key aspects of working in an interpersonal profession is fostering relationships and connections with others, which is especially crucial for ECTs. Peer mentoring, social development, and professional conversations are vital in the early career period [35,38,39].

The findings were consistent with the pilot study in that the weekly reflections (e.g., benefits experienced) could be linked to the reported change in attention awareness (MAAS), perceived stress (PSS), and subjective wellbeing (PWI) [72]. The participant reflections (benefits experienced and session description) suggested a change in perceived stress. The inclusion of cognitive-behavioural mechanisms supports the ability to regulate through reframing and reappraisal [11], and increased body awareness provides the opportunity to recognise the stress response (physically) and implement techniques to decrease the stress response (e.g., practicing breathing techniques when feeling stressed). For instance, the participants shared that they felt a decrease in the stress response and an increased sense of calm (e.g., feeling less “heightened”). Feeling calmer and at ease indicates a decrease in reactive (habitual) response to events or situations [11]. The participants felt “refreshed” and enjoyed having the opportunity to “unwind” and “relax”. In addition, the session descriptions highlighted that the physical component provided “stress reduction”, “relaxation”, “calm”, and decreased “muscle tension”, which may relate to the decrease in salivary cortisol levels for the pre- and post-session samples. Similarly, the reported benefits regarding improved sleep (quality and duration) may relate to the changes in cortisol levels (e.g., CAR) experienced by the participants.

Changes in body awareness, increased awareness of breathing, and the stress response were reported benefits that corresponded to the change in present moment awareness (MAAS scores). Awareness of sensations in the body and thoughts cultivated through stress management techniques (e.g., attention stability, response inhibition) can reduce negative reappraisal and rumination [50], and this was evidenced in the reported benefits (Figure 3). Mood changes have been associated with increased body awareness (e.g., Hatha yoga) [89].

The participant post-session feedback (session description) may support the change in the subjective wellbeing (PWI). The PWI is discussed with reference to the dimensions of wellbeing [29]. The participants shared that the program was “informative,” “relevant,” and “interesting” and created the opportunity to learn strategies and techniques to support their wellbeing. It could be suggested that gaining or learning new information relates to the dimension of wellbeing: *personal growth*. Similarly, the participants identified the connection between teacher functionality and wellbeing and increased awareness of behaviours and self-realisation, which supports the dimensions of *autonomy* [29]. Providing individuals with techniques to decrease stress and promote wellbeing effectively creates behaviour changes that promote wellbeing [50] and increase autonomy. The participants shared that they were about to identify patterns in their behaviour and decrease rumination, negative reappraisal of situations, and reactive behaviour through implementing techniques and strategies.

Meditation practices operate through the mechanism of attention stability, concentration, and self-realisation (meta-awareness) [50,51,52], which may support the dimension of wellbeing: *personal growth.* Ryff and Singer [29] refer to *personal growth* as the continual development of self-realisation throughout one’s lifespan. In addition, *self-acceptance* is the dimension of wellbeing that is central to mental health and optimal functioning and refers to the attitudes one holds towards oneself [29]. Maintaining present-moment attention, engagement, and interoceptive and proprioceptive awareness of oneself and others (ethical principles–behaviour) are present in all of the techniques and mechanisms included in the physical component of the intervention (based on the system of yoga) [44,49,50,93]. Similarly, practicing non-reactive awareness and self-compassion are techniques that increase self-acceptance.

The findings suggest that techniques that reduce the stress response operate through underlying mechanisms that promote the dimensions of wellbeing. The inclusion of participant reflections suggested that the techniques and mechanisms (physiological and psychological) created the opportunity for behavioural changes that promoted wellbeing. The results warrant further investigation with a larger participant sample or a longitudinal study design to determine the ongoing benefits of CIs for educators.

## 5. Conclusions

The focus of the present study was to explore the mechanisms for promoting integrated (holistic) wellbeing, particularly the relationship between the stress response (physiological and psychological), stress management techniques (mechanisms), and subjective wellbeing. The convergent mixed-methods design underpinned by a dialectic perspective provided insight into the participants’ lived experiences in the program. The findings highlighted the application of the techniques and underlying mechanisms in professional contexts, supporting the findings presented in existing qualitative studies for MBIs and CIs for educators. A unique finding from the present study related to the benefits experienced by the participants and the application of the techniques in personal contexts. The findings may support the inclusion of CIs for educators to support induction processes and promote wellbeing in schools more broadly.

## Figures and Tables

**Table 1:** Participant relaxation activities (*n* = 24).

Social	Physical	Personal	Ambiguous	No Activities
Dinner or coffee with friends (weekly) (*n* = 4) Walking with friends (*n* = 2)	Walking (*n* = 7) Exercise (*n* = 4)	Reading (*n* = 5) Listening or playing music (*n* = 2) Craft and puzzles (*n* = 1) Bath (*n* = 2)	Watching television (*n* = 5) C*i*nema (*n* = 4) YouTube (*n* = 2)	(*n* = 0)

**Table 2:** Summary of participant response examples from weeks 2 to 6.

Awareness	Example Participant Response		
Self	Realising my mood affects people around me (Jo, week 3).	Noticing when my thoughts are racing and when I’m not being present (Rachel, week 3).	Teaching others about breathing, more self-regulation myself and aware of my breathing and my emotions (Jess, week 4).
Breathing	I have been focusing on my breathing whenever I feel anxious or stressed about something (Kelly, week 2).	When angry, I was able to go do some breathing exercises to help me calm down (Rachel, week 4).	Deep breathing and yoga is helping me concentrate and focus on the task at hand (Anthony, week 5).
Body awareness	I’m feeling more aware of where I hold stress in my body, and I’m trying to take time (a minute) to stop and breathe (Beth, week 2).	More aware of body reactions and impact of external influences on internal moods (Victor, week 5).	More aware of body feelings and emotions (Sonya, week 6).
Behaviour	I am thinking a lot more of my sleeping and eating patterns. Trying to walk more. Trying to take 10 min each night to listen to relaxing music (Sarah, week 2).	Making time for relaxing activities and yoga (Sonya, week 4).	Thinking more about personal health through diet. Thinking more carefully about food choices (Victor, week 6).

**Table 3:** Events identified by the participants as either positive, negative, or neutral (*n* = 24).

Week	Positive	Negative	Neutral	No Response
1	7	11	10	0
2	11	9	8	1
3	9	9	11	0
4	5	8	11	2
5	6	10	8	1
6	8	6	10	1

**Table 4:** Participant weekly reflection negative events.

Domain.	Theme	Responses *(n* = 52)		Sub-Theme	Example Participant Response
Weekly event		*n*	%		
	Family	3	17%	Health	Partner has been unwell, so I have felt stressed from having to do everything.
		6		Relationship	
	Work	6	65%	Workload	A couple of set-backs career related. I have had issues with colleagues overstepping… and an argument with a colleague. Escalating student behaviour with little support from admin. Time management worries and external requirements for reporting.
		16		Student behaviour	
		7		Colleague interaction	
		3		Student parent interaction	
		2		Observations/performance	
	Personal	6	17%	Relationships	Breakup issues with an ex-partner who is rude and aggressive. Feeling unwell with a head cold/sinus infection.
		3		Health concern	

**Table 5:** Participant weekly reflection positive events.

Domain.	Theme	Responses (*n* = 31)		Sub-Theme	Example Participant Response
Weekly event		*n*	%		
	Family	6	29%	Family time or events	Positive family time with my children over the weekend. Son’s volleyball club team won the State championships.
		3		Children	
	Work	3	35%	Student success	Working with students and seeing a positive result, meeting with people, and hearing positive feedback on a student.
		8		Observations/performance	
	Personal	1	35%	Personal relationships	Super-engaged students secured a job interview. Getting together with friends for a birthday celebration. Had time to go for several walks, spent time with friends I don’t often see. More me-time.
		4		Friends	
		6		Extra time	

**Table 6:** Summary of self-report measures for Ashley pre-and post-program and 3-month follow-up.

Measures	Week 0	Participant Group Mean	Week 6	Participant Group Mean	Change	3-Month Post-Program
PSS	19	21.13	16	16.63	−3	11
MAAS	2.93	3.55	4.13	4	1.2	3.87
PWI	43	52	59	57.4	16	53

**Table 7:** Summary of self-report measures for Sarah pre-and post-program and 3-month follow-up.

Measures	Week 0	Participant Group Mean	Week 6	Participant Group Mean	Change	3-Month Post-Program
PSS	24	21.13	12	16.63	−12	11
MAAS	2.8	3.55	4.2	4	1.4	4.67
PWI	46	52	68	57.4	22	64

## Data Availability

Data available on request due to restrictions. The data are not publicly available due to the conditions specified in the ethics application.
